# Polystyrene Nanoplastics Induce DNA Damage and Excitotoxicity in Whole-Brain Organoids: The Role of the TLR9/MyD88 Pathway

**DOI:** 10.3390/toxics14010005

**Published:** 2025-12-19

**Authors:** Yizhe Wei, Gaofang Cao, Jianping Ma, Yanan Mi, Yiming Zhao, Leili Zhang, Bingyan Wang, Huanliang Liu, Kang Li, Yue Shi, Wenqing Lai, Lei Tian, Bencheng Lin

**Affiliations:** 1 School of Public Health, Binzhou Medical University, Yantai 264003, China; 2 Military Medical Sciences Academy, Academy of Military Sciences, Tianjin 300050, China

**Keywords:** polystyrene nanoplastics, whole-brain organoids, model of early exposure, TLR9, neurodevelopmental toxicity

## Abstract

Polystyrene nanoplastics (PS-NPs) can cross the placenta and blood–brain barrier to accumulate in the fetal brain following inhalation or ingestion, raising concerns about PS-NPs-induced developmental neurotoxicity (DNT). However, current evidence regarding the mechanisms underlying PS-NPs-elicited DNT remains critically scarce. Given the inherent limitations of two-dimensional cell culture techniques, we employed a whole-brain organoid (WBO) model, which more faithfully recapitulates the dynamic changes and substantial alterations during the early development of the human nervous system, to investigate the PS-NPs-induced DNT. Developing WBOs were exposed to 50-nm PS-NPs at concentrations of 50 and 100 μg/mL. Additionally, we established an early developmental exposure model in neonatal rat for robust validation. The results revealed aberrant formation of the tissue architecture of neural epithelial buds in PS-NPs-exposed WBOs, accompanied by significant inflammatory responses and oxidative stress. Marked DNA damage and substantial activation of the TLR9/MyD88 pathway were observed in WBOs and in the cerebral cortex of neonatal rat, leading to significant upregulation of the excitotoxicity marker c-Fos and the excitatory synaptic marker NMDAR. In vitro assays revealed that melatonin treatment could efficiently counteract PS-NPs-mediated neuronal impairment, with both the reduced cell viability and excessive DNA damage induced by PS-NPs being restored to levels close to those of the control group. In conclusion, by establishing WBOs and early developmental exposure models in neonatal rat, we found that PS-NPs can induce DNA double-strand breaks, and activation of the TLR9 pathway mediates PS-NPs-induced excitotoxicity.

## 1. Introduction

Polystyrene nanoplastics (PS-NPs) are prevalent in drinking water; the atmosphere; foods including seafood, salt, honey, and sugar; and beverages such as beer and tea [1,2]. In recent years, the presence of NPs has been reported in a variety of human tissues, organs, and body fluids [3,4,5]. Studies have found that environmental concentrations of PS-NPs exceed those of microplastics, and PS-NPs with smaller particle sizes more readily permeate cell membranes, causing stronger toxic effects [6]. Numerous studies have observed that NPs can cross the placental barrier and blood–brain barrier [7], and they are heavily enriched in the brain, which contains 30-fold more microplastics than liver and kidney samples [8]. Studies on the effects of PS-NPs on brain development have identified four key components: ingestion and bioaccumulation in the food chain [9], translocation across biological barriers, induction of oxidative stress and inflammation [10], and endocrine disruption [6].

Although some studies have suggested that PS-NPs can adversely affect neurodevelopment [7], reports on this topic remain limited. Moreover, in-depth investigations into the ability of PS-NPs to induce neurotoxicity during embryonic development are particularly lacking, and the underlying mechanisms remain unclear. Most existing studies relied on experimental evidence from animal models, which can exhibit biological differences that affect their predictive utility and extrapolation to human conditions. By contrast, whole-brain organoids (WBOs) derived from human induced pluripotent stem cells (hiPSCs) more closely resemble the structure of the human fetal brain in terms of cellular composition, gene expression profiles, and protein composition [11,12,13]. For instance, recent research has demonstrated that WBOs can self-organize and expand in vitro while retaining the regional identity and developmental characteristics of the human fetal brain. As such, WBOs represent a more accurate model for studying human brain development and associated diseases than traditional animal models. Because of ethical limitations, experiments cannot be performed during the actual process of human organogenesis. Therefore, WBO technology has emerged as the optimal alternative for neurodevelopmental research. For instance, Chena [14] and Huang [15] utilized organoids to investigate the mechanisms of microplastic neurotoxicity.

The DNA damage response (DDR) plays a critical role in the nervous system, affecting the maintenance and function of long-lived neurons in the adult brain. In embryonic tissues, the early stages of neurogenesis are highly sensitive to DNA damage [16]. Defects in DNA repair mechanisms are associated with neurodevelopmental disorders, such as neurodevelopmental defects and microcephaly [17]. However, the effects of PS-NPs on DDR during human neurodevelopment remain unreported.

Toll-like receptors (TLRs) are type I transmembrane glycoproteins and part of the pattern recognition receptor family [18]. TLR9 is an innate immune sensor that recognizes intracellular double-stranded DNA. Research has found that nanoplastics can cause cognitive and memory impairments, epilepsy, and anxiety-like behaviors in laboratory animals, although the underlying mechanisms remain unclear. This study provides insights into the mechanism by which PS-NPs induce these adverse effects through the TLR9/MyD88 signaling pathway.

## 2. Materials and Methods

### 2.1. WBO Modeling

hiPSCs (GD0000l, Guidon Pharmaceutics, Beijing, China) were cultured at 37 °C in a 5% CO_2_ incubator (Thermo Fisher Scientific, Waltham, MA, USA). hiPSCs were cultured according to a previously reported protocol [19]. Following the instructions of the brain organoid differentiation kit (Cat No.: GDK012, Guidon Pharmaceutics, Beijing, China), differentiation was induced when the cell confluence reached 80% and the percentage of heterogeneous cells was less than 10%. The culture medium was changed every other day, changing half of the original medium volume each time, and the diameter and development of WBOs were observed each time. After reaching approximately 500–600 µm in diameter, the EBs were transferred to a low-attachment 24-well plate (FULA243-5pcs, Beyotime Biotechnology, Suzhou, China) containing 500 µL of nerve induction medium. After transferring neuroepithelial tissue to Matrigel droplets, the culture was continued in the CO_2_ incubator. The detailed procedure follows Lancaster’s method for constructing WBOs [19].

### 2.2. PS-NPs Characterization and Exposure Method

PS-NPs (50-nm PS-NPs suspension, PS000050, density = 1.05 g/mL) were purchased from Beijing Zhongkelaiming Technology Co., Ltd. (Beijing, China). Our previously reported characterization of PS-NPs confirmed their spherical shape with an average diameter of approximately 50 nm. Dynamic light scattering analysis in ultrapure water indicated that the hydrodynamic diameter of PS-NPs was 56.71 ± 25.54 nm, with a maximum size of 64.20 nm [7]. Exposure concentrations (50 and 100 µg/mL) were selected on the basis of published environmental and brain-organoid studies [14,15,20]. On day 12 of WBO culture, following stabilization of the differentiation phase, interventions with PS-NPs were conducted at concentrations of 50 and 100 µg/mL. Meanwhile, the control group was maintained under normal culture conditions. The culture medium was replaced every 3 days, and the growth status, diameter, and perimeter of WBOs were monitored.

### 2.3. Whole-Transcriptome Sequencing

Total RNA was extracted using the TRIzol method. Tissue samples were ground in liquid nitrogen and transferred to 1.5-mL centrifuge tubes. One milliliter of TRIzol reagent (Thermo Fisher Scientific) was added, followed by vigorous shaking. The mixture was incubated at room temperature for 5 min to dissociate nucleoprotein complexes. Subsequently, 200 μL of chloroform were added, and the sample was shaken vigorously for 15 s and centrifuged at 12,000 rpm and 4 °C for 15 min. The upper aqueous phase was transferred to a new tube, and an equal volume of phenol:chloroform (25:24) was added. After vigorous shaking, the sample was centrifuged again at 12,000 rpm and 4 °C for 15 min. The upper aqueous phase was collected, and an equal volume of isopropanol was added, followed by incubation at −20 °C for 1 h. The sample was centrifuged at 12,000 rpm and 4 °C for 10 min to precipitate the RNA. The supernatant was removed, and the RNA pellet was washed with 1 mL of 75% ethanol. The sample was centrifuged at 8000 rpm and 4 °C for 5 min to remove residual ethanol. The RNA pellet was air-dried for 5–10 min and dissolved in 20–50 μL of RNase-free water. The RNA solution was incubated at room temperature for 10 min, vortexed, and briefly centrifuged to ensure complete dissolution. The RNA was stored at −80 °C for further use.

RNA quality was assessed using a NanoDrop One spectrophotometer (Thermo Fisher Scientific) by measuring the A260/A280 ratio, indicating RNA purity. RNA integrity was evaluated using an Agilent 2100 Bioanalyzer (Agilent Technologies, Santa Clara, CA, USA) through capillary electrophoresis. Only RNA samples with a RIN greater than 7.0 were used for downstream applications. RNA sequencing was performed by Gene Denovo Biotechnology Co., Ltd. (Guangzhou, China) using the Illumina NovaSeq X Plus platform (Illumina, San Diego, CA, USA). The extracted RNA was used to construct cDNA libraries, which were then subjected to quality control to ensure high-quality sequencing data. The libraries were loaded onto the Illumina NovaSeq X Plus for high-throughput sequencing, generating paired-end reads with a read length of 150 bp. Bioinformatics analysis was performed using Omicsmart, a dynamic real-time interactive online platform for data analysis (http://www.omicsmart.com, accessed on 19 June 2025). The R package(version 4.4.1) (The R Foundation for Statistical Computing, Vienna, Austria) pheatmap and the platforms for Gene Ontology (GO) analysis, Kyoto Encyclopedia for Genes and Genomes analysis, and gene set enrichment analysis (GSEA) were all conducted using Omicsmart. For differential gene expression analysis following sequencing, the threshold was set using edgeR with the criteria of |log2FC| > 1 and FDR < 0.05. In GSEA, the filtering criteria were *p* < 0.05, FDR < 0.25, and an absolute value of the normalized enrichment score greater than 1.

### 2.4. PS-NPs Exposure and Melatonin Treatment of Neuronal Cells

Neuronal cells (CP-H122, Procell, Wuhan, China) were exposed to 50-nm PS-NPs for 48 h. Exposure concentrations (50 and 100 µg/mL) were selected on the basis of published environmental and studies [14,15,20]. The exposure concentrations were set as 0, 25, 50, and 100 μg/mL, with each concentration corresponding to a separate experimental group.

In this study, some experimental groups received additional treatment during PS-NPss exposure. Melatonin (HY-B0075, MedChemExpress, Monmouth Junction, NJ, USA) was dissolved in DMSO to prepare a 10-mM stock solution. The stock solution was stored at −20 °C in the dark. Before use, the stock solution was diluted with cell culture medium to the working concentration. All operations were performed strictly in accordance with the manufacturer’s instructions.

### 2.5. ELISA

Conditioned medium was collected from WBOs at the maturation stage and analyzed for TNF-α (TNF-α ELISA Kit, MM-0122H1, Jiangsu Enzyme Free Industry Co., Ltd., Liyang City, China), IL-1β (Human IL-1β ELISA Kit, MM-0181H1, Jiangsu Enzyme Free Industry Co., Ltd., China), and catalase (CAT) levels (Human CAT ELISA Kit, MM-12716H1, Jiangsu Enzyme Free Industry Co., Ltd.) according to the manufacturers’ protocols.

After neuronal cells were collected, 8-hydroxy-2′-deoxyguanosine(Human 8-Hydroxy-2′-deoxyguanosine (8-OHdG) ELISA Kit,CB10037-Hu,COIBO BIO, China), serotonin levels (Human 5-HT ELISA Kit, CB10030-Hu, COIBO BIO, China), LDH (CYQUANT™ LDH Cytotoxicity Assay Kit, C20300, Thermofisher, USA) and MDA (Micro Malondialdehyde (MDA) Assay Kit (TBA Method), A003-2, Nanjing Jiancheng Bioengineering Institute, Jiangsu, China) were analyzed in accordance with the manufacturer’s protocol. Monoclonal antibodies were added to the microplates (464718, Thermo Fisher Scientific, Waltham, MA, USA). After incubation, horseradish peroxidase (HRP)-conjugated detection antibodies were added to form a sandwich immunoassay. Substrate solution was then added, and HRP catalyzed the substrate to produce a color change. Stop solution was subsequently added to terminate the enzyme reaction, and the absorbance was measured at 450 nm within 15 min.

### 2.6. Immunofluorescence

WBOs were placed in PBS containing 2% sucrose, and after embedding in OCT (G6059, Servicebio, Wuhan, China), organoids were placed in liquid nitrogen for rapid freezing. WBO samples were sectioned on a frozen microtome (CM1950, Leica Biosystems, Nussloch, Germany) at a thickness of 10–15 µm. Frozen sections were affixed to precooled slides (catalog No.: 80821,ibidi GmbH, Martinsried, Germany). After overnight incubation at 4 °C with the primary antibody, the samples were washed with PBS containing 0.1–0.3% PBST. The organoids were then incubated with secondary antibody at room temperature in the dark for 3 h. The antibodies are listed in Table 1. Following antibody exposure, WBOs were observed using a sweeper (PANNORAMIC SCAN I, 3DHISTECH, Budapest, Hungary). ImageJ software (Version 1.54r, US National Institutes of Health, Bethesda, MD, USA) was used to quantify the fluorescence intensity of each image.

### 2.7. Establishment of an Early Developmental Exposure Model in Neonatal Rats

Pregnant Sprague Dawley rats were purchased from SBF Biotechnology Company (Beijing, China). All rats were individually housed in a specific pathogen-free facility maintained on a 12-h/12-h light/dark cycle with controlled temperature (22 °C ± 2 °C) and humidity (40–50%). Pregnant rats were randomly assigned to the experimental group, which received PS-NPs via gavage administration during pregnancy and lactation (PS-NPs exposure dose of approximately 2.5 mg/kg/day), or the control group, which was administered ultrapure water by gavage (3 dams/group). After natural delivery, all offspring were reared for subsequent experimental assays. On the 22nd day after weaning (PND22), the cerebral cortex of each pup was collected for subsequent multi-stage neurodevelopmental studies. This study was approved by the Ethics Committee of Tianjin Institute of Environmental Medicine and Operational Medicine (Ethics number: IACUC of AMMS-04-2022-015).

### 2.8. Western Blotting

Proteins were extracted from the neonatal rat cortex and cultured with human-derived neuronal cells using a mixture of protease and phosphatase inhibitors and RIPA lysis buffer. Protein concentrations were quantified using a BCA protein quantification test kit (PC0020, Solarbio, Beijing, China). Subsequently, proteins were separated by SDS-PAGE using gel preparation reagent (P1200, Solarbio) and subsequently transferred to a membrane. The membrane was blocked with 5% skim milk at room temperature for 60 min, followed by overnight incubation at 4 °C with primary antibodies against γH2AX (1:1000, AB303656, Abcam, Cambridge, UK), TLR9 (1:1500, AF8193, Cell Signaling Technology, Danvers, MA, USA), MyD88 (1:25,000, 23230-1-AP, Proteintech, Rosemont, IL, USA), c-Fos (1:1000, YM3469, Immunoway, San Jose, CA, USA), and NMDAR (1:500,AF6406, Affinity Biosciences, Jiangsu, China). After incubation with the primary antibodies, the membrane was to HRP-conjugated goat antirabbit or goat antimouse secondary antibody at room temperature for 1 h. Finally, the membrane was incubated with the ECL reagent kit (Beyotime Biotechnology, Haimen, China) at room temperature for 2 min, and the signal intensity was captured using a chemiluminescence apparatus (MINI-CHEMI, JUNYI, Beijing, China). Band intensity was analyzed by the grayscale software Gelpro32 (Tanon, Shanghai, China), using tubulin or β-tubulin as an internal reference for protein quantification and normalization.

### 2.9. Statistical Methods and Plotting

We used an independent-samples *t*-test to assess the statistical differences between the two datasets. One-way analysis of variance was conducted to evaluate distinctions among multiple groups, followed by Tukey’s multiple comparison test for intergroup disparities arising from distinct treatments. The analysis was performed using IBM SPSS Statistics 25 (IBM, Armonk, NY, USA) and GraphPad Prism 9.0 (GraphPad Software, Inc., Boston, MA, USA). The results were expressed as the mean ± SD. The arrows and scale bars in the figures were added using ImageJ software. The cartoon images were drawn using FigDraw (www.figdraw.com).

## 3. Results

### 3.1. WBO Developmental Identification and Trajectory Characterization

**Figure 1:**
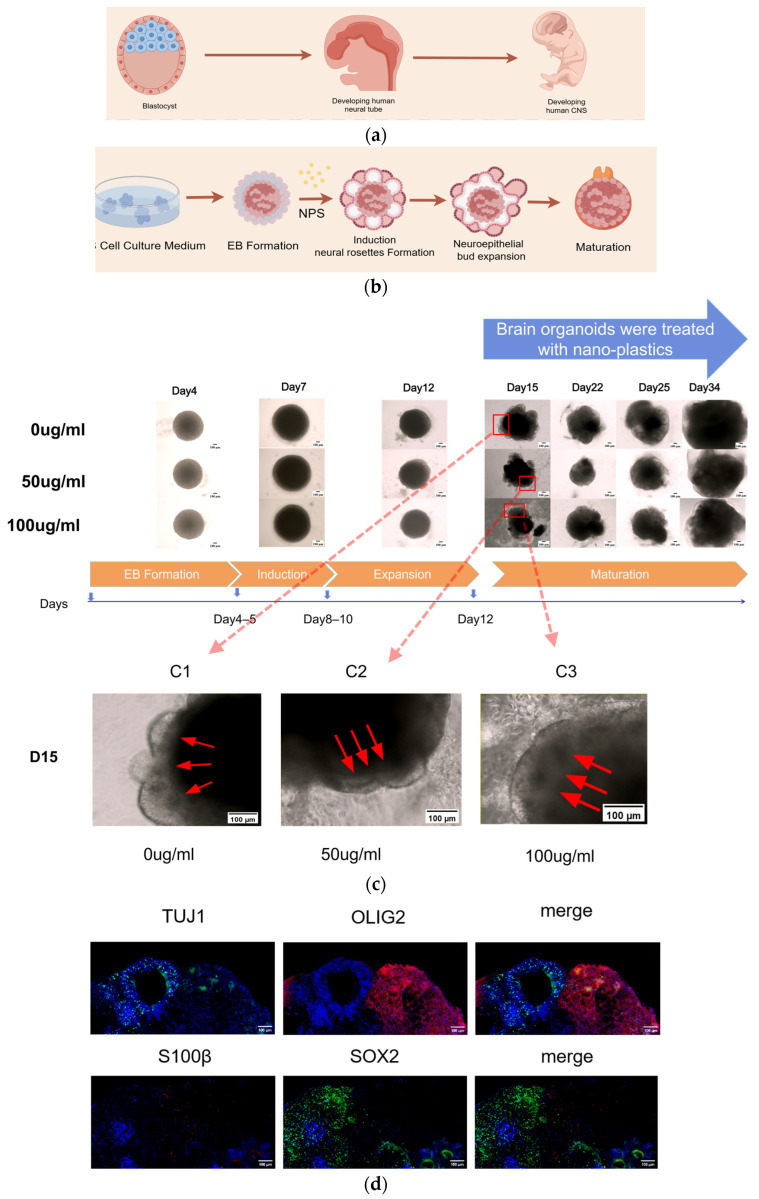
Differentiation of human embryonic stem cells into WBOs. (**a**) Schematic diagram of the differentiation process of iPSCs into brain. (**b**) Characteristic changes experienced by HiPSCs during the development into WBOs. (**c**) Morphological changes and exposure timing of WBOs at different developmental stages (EB formation, Induction, Expansion and Maturation). The area marked by the red arrow is the neural ectoderm bud. (**d**) Identification of cell types in WBOs. Immunohistochemical analysis of neural progenitor cells (SOX2), neurons (TUJ1), oligodendrocytes (OLIG2), and astrocytes (S100β) after 40 days of culture. Scale bar = 100 µm (**c**,**d**).

In humans, neural tube formation begins 3 weeks after fertilization (Figure 1a). Ectodermal cells form a neural plate structure early in embryonic development. Subsequently, the two edges of the neural plate thicken and fold upward to close and form the neural tube. The neural tube is the primordium of the central nervous system, and it differentiates into the brain and spinal cord, as well as the pineal gland, pituitary gland, and retina (Figure 1a) [21]. In vitro, the neural tube that arises in WBOs is known as the neural rosette (Figure 1c) [22,23]. As WBOs mature, SOX2 expression in neural progenitor cells, TUJ1 expression in the neuronal layer, S100β expression in astrocytes, and OLIG2 expression in oligodendrocytes are detected (Figure 1d).

In this study, WBO development was divided into four phases: EB formation, induction, expansion, and maturation. The WBOs at each of these stages were observed using microscopy (Figure 1c). On day 15 of culture, healthy cell aggregates of WBOs exhibited smooth edges. The neuroepithelium developed on the outer surface, forming optically translucent neuroectodermal buds with a transparent radial organization. During the expansion phase, WBOs formed a dense core and developed a distinct laminar structure (Figure 1c, C1).

Conversely, WBOs exposed to 50 µg/mL PS-NPs lacked a distinct laminar structure compared with that in the control group. The stratification of the cortex was less evident, and the number of neuroectodermal buds was lower than that in controls (Figure 1c, C2). WBOs exposed to 100 µg/mL PS-NPs exhibited poor development. The neuroectodermal buds in this group were not distinctly laminar, and the light–dark layering was absent. Additionally, these WBOs lacked optical clarity and contained a large amount of cell debris (Figure 1c, C3).

### 3.2. PS-NPs Induce Inflammatory Responses and Oxidative Stress in WBOs,, and Exert Cytotoxic Effects on Neuronal Cells

**Figure 2:**
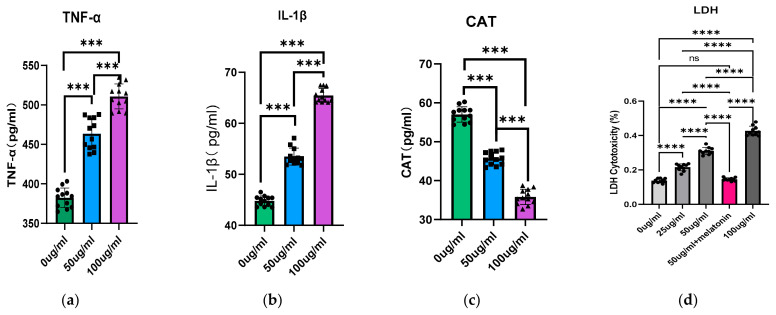
(**a**–**c**) Expression changes in inflammatory cytokines (IL-1β, TNF-α) and oxidative stress marker (CAT) in WBOs Culture Medium after PS-NPs exposure (*n* = 12). (**d**) LDH assay of cultured neurons in vitro following PS-NPs exposure and melatonin intervention (*n* = 10). Symbols such as ▲, ■, ● represent individual samples within each group. (statistical significance was determined by ANOVA (SPSS 25.0, IBM Corp, Armonk, NY, USA) followed by Tukey’s multiple comparisons test, with, *** *p* < 0.001 and **** *p* < 0.0001, ns indicate no significant difference).

Under PS-NPs exposure, WBO exhibited significant inflammatory responses and oxidative stress in a concentration-dependent manner. Compared with the results in the control group, PS-NP-exposed WBOs displayed a prominent inflammatory response, with significantly elevated levels of IL-1β and TNF-α (both *p* < 0.05, Figure 2a,b). PS-NPs-exposed WBOs also exhibited obvious oxidative stress, characterized by a significant decrease in CAT levels (*p* < 0.05, Figure 2c).

Under PS-NPs exposure conditions, neuronal cells exhibited significant cytotoxicity. Compared with the control group, PS-NPs-treated neuronal cells showed remarkable cytotoxic damage, which was specifically manifested by a significant increase in LDH leakage levels in the culture medium (*p* < 0.05) (Figure 2d).

### 3.3. PS-NPs Exposure Induces DNA Damage and Activates the TLR9 Pathway, Thereby Mediating Excitotoxicity

**Figure 3:**
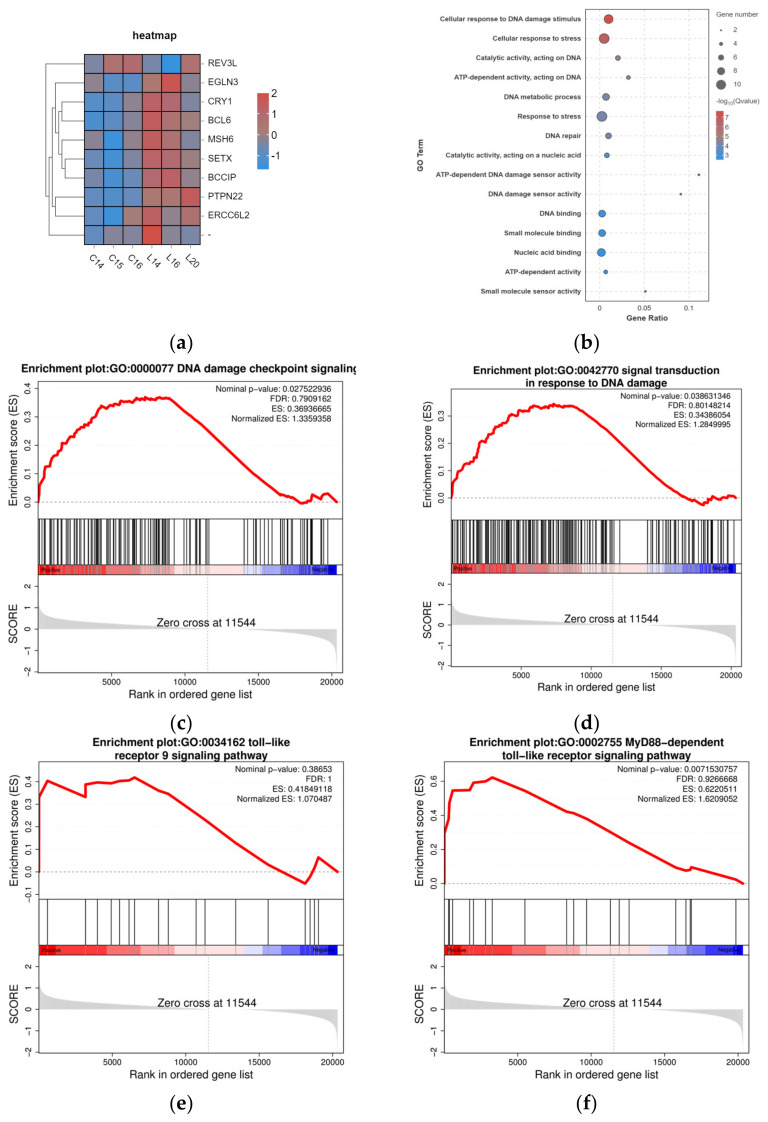
WBOs whole-transcriptome sequencing. (**a**) Heatmap of DNA damage repair mRNA differences. (**b**) Gene Ontology (GO) Annotations for DNA Damage Repair and Toll-like Receptor (TLR) Pathways. (**c**) The GSEA reveals the enrichment of the DNA damage checkpoint signaling pathway (GO:0000077). (**d**) The GSEA reveals the enrichment of signal transduction in response to DNA damage (GO:0042770). (**e**) The GSEA reveals the enrichment of Toll-like receptor 9 signaling pathway (GO:0034162). (**f**) The GSEA reveals the enrichment of MyD88-dependent Toll-like receptor signaling pathway (GO:0002755). (**c-f**) Middle vertical lines (red/blue): Indicate the positions of genes belonging to the signaling pathway in the ranked gene list. Vertical dashed line: Marks the “zero cross” in the ranked gene list, representing the boundary where the gene-phenotype correlation shifts between positive and negative.

Previous studies demonstrated that reactive oxygen species (ROS) generated following oxidative stress induce oxidative DNA damage, including DNA strand breaks [24]. Our whole-transcriptome sequencing analysis of WBOs revealed that genes associated with DNA damage repair were significantly upregulated, as evidenced by the heatmap of differentially expressed genes (Figure 3a). Notably, GO analysis revealed significant enrichment in cellular response to DNA damage stimulus (GO: 0006974) and DNA repair (GO: 0006281; Figure 3b). Furthermore, GSEA revealed significant enrichment in several pathways, including “DNA damage checkpoint signaling,” “Signal transduction in response to DNA damage,” “Toll-like receptor 9 signaling pathway,” and “MyD88-dependent toll-like receptor signaling pathway” (Figure 3c–f). The DNA damage checkpoint signaling pathway plays a pivotal role in pausing the cell cycle upon DNA damage occurrence, thereby permitting subsequent repair or rectification processes [25]. The significant enrichment of the DNA damage checkpoint signaling pathway indicates that this pathway plays a critical role in the toxic mechanism induced by PS-NPs. The “Signal transduction in response to DNA damage” pathway describes the cascade of intracellular signaling events initiated upon the detection of DNA lesions. Research has demonstrated that histone H2AX is rapidly recruited to sites of DNA damage, where it plays a critical role in the activation of downstream repair mechanisms [26]. The enrichment of this pathway, as revealed by GSEA, suggests that exposure to PS-NPs induces DNA damage, triggering the associated signaling cascades.

**Figure 4:**
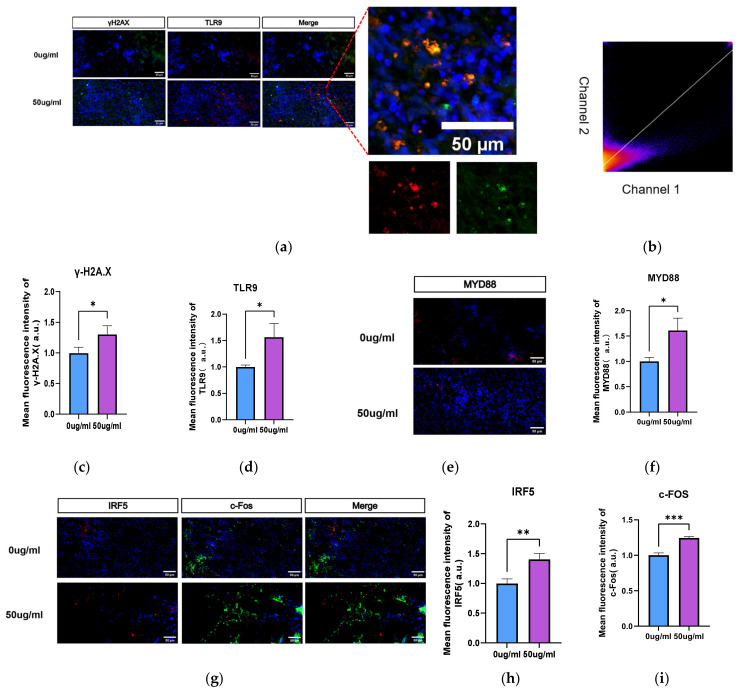
Exposure to PS-NPs induces significant alterations in TLR9 indicators with in WBOs. (**a**) Representative immunofluorescence images of labeled DNA damage of γH2A.X (green); Representative immunofluorescence images of labeling of TLR9 (red) (magnified region is colocalization of TLR9 and DNA damage). (**b**,**c**) Immunofluorescence intensity of quantified γH2A.X, TLR9. (**d**) 2D intensity histogram. The color depth of each point in the figure represents the frequency of pixel occurrence under the combination of the (Ix, Iy) intensity values, and the darker the color, the higher the frequency. Pearson’s R value (no threshold) = 0.71. (**e**) Representative immunofluorescence images of the labeling of My88 (red). (**f**) Quantitative immunofluorescence intensity of MyD88. (**g**) Representative immunofluorescence images of the labeling of IRF5 (red); Representative immunofluorescence images of the labeling of c-FOS (green). (**h**,**i**) Quantification of immunofluorescence intensity of IRF5, c-Fos. Compared with controls, * *p* < 0.05, ** *p* < 0.01, *** *p* < 0.001. The fluorescence intensity was quantified and normalized as arbitrary units (*a.u.*). The bar graphs represent the normalized quantification results. Statistical analysis was performed using an independent samples *t*-test.

**Figure 5:**
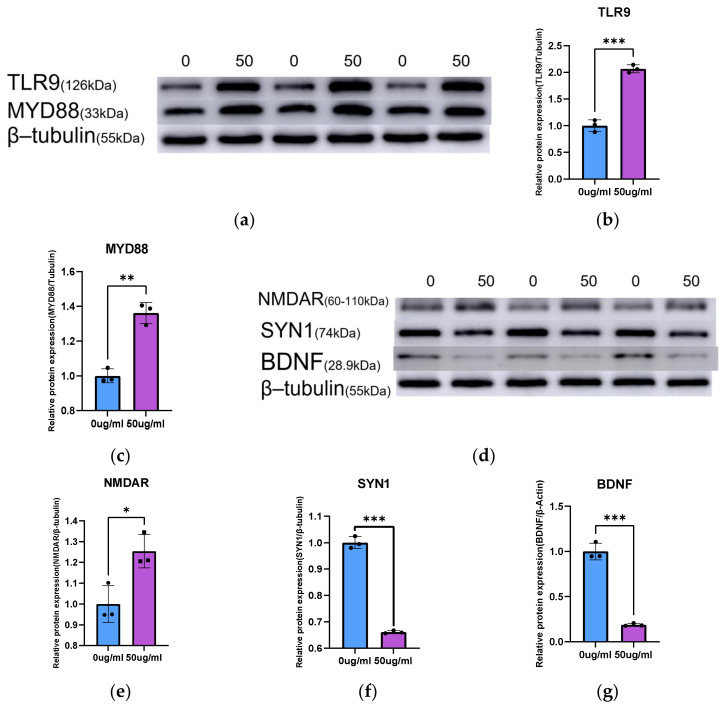
TLR9 pathway activation in rat cortex. (**a**) Western blotting to detect TLR9, MyD88 in cortical tissue; β-Tubulin as the internal reference protein (**b**,**c**) Quantified TLR9, MyD88 expression. (**d**) Western blotting to detect BDNF, NMDAR, and SYN1 in cortical tissues; β-Tubulin was used as the internal reference protein. (**e**–**g**) The quantitative analysis of NMDAR, SYN1 and BDNF. * *p* < 0.05, ** *p* <0.01, *** *p* <0.001. The bar graphs represent the normalized quantification results. Symbols such as ■, ●represent individual samples within each group. Statistical analysis was performed using an independent samples *t*-test.

Immunofluorescence experiments further validated the accuracy of the transcriptome sequencing results. γH2A.X and TLR9 expression was significantly elevated in WBOs exposed to PS-NPs (Figure 4a–c). Quantification of fluorescence co-localization revealed a strong positive correlation between the fluorescence intensities of γH2A.X and TLR9 (Pearson correlation coefficient = 0.71; Figure 4d). Following PS-NPs exposure, the protein expression of TLR9 and MyD88 in WBOs was significantly increased (both *p* < 0.05; Figure 4); similarly, the expression of TLR9 and MyD88 was also significantly elevated in the cerebral cortical tissues of neonatal rat (Figure 5a–c).

To verify the occurrence of excitotoxicity in WBOs, we detected the excitotoxicity marker c-Fos. The results demonstrated that c-Fos expression was significantly increased in both WBOs (both *p* < 0.05; Figure 4g,i). Excitotoxicity disrupts the balance between excitatory and inhibitory synapses, leading to abnormal neural activity. Western blotting illustrated that NMDAR expression was significantly increased in the cerebral cortex of neonatal rat (Figure 5d,e), whereas that of SYN1 (Figure 5d,f) and BDNF (Figure 5d,g) was significantly decreased.

### 3.4. Supplementation with Melatonin Can Reverse DNA Damage and TLR9 Pathway Activation Induced by 50 μg/mL PS-NPs in Neurons

**Figure 6:**
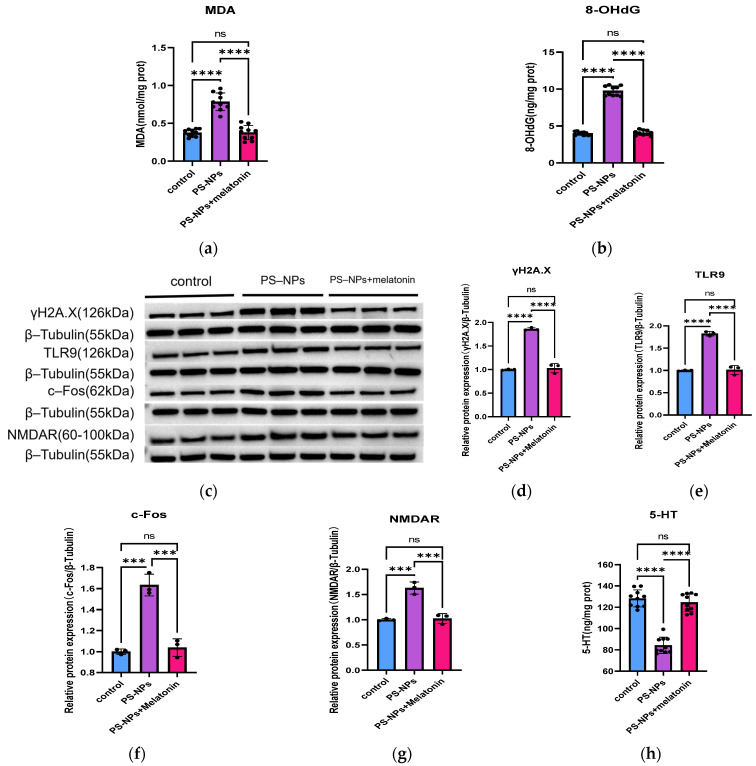
Modulation of the TLR9 pathway in neurons following melatonin intervention. (**a**) Quantitative analysis of malondialdehyde (MDA) levels in neurons (*n* = 10); (**b**) Quantitative analysis of 8-hydroxy-2′-deoxyguanosine (8-OHdG) levels in neurons (*n* = 10); (**c**) Western blot analysis of γH2A.X, TLR9, c-Fos, and NMDAR in neurons (*n* = 4), with β-Tubulin serving as the loading control; (**d**–**g**) Quantitative analysis of γH2A.X, TLR9, c-Fos, and NMDAR expression; (**h**) Quantitative analysis of 5-HT levels in neurons (*n* = 10); Compared with the control group, *** *p* < 0.001, **** *p* < 0.0001; “ns” indicates no statistically significant difference. Individual symbols (●) represent single samples within each group. Quantitative results were normalized, and data in bar graphs are presented as M ± SD.

To verify that PS-NPs exposure significantly induces DNA damage and activates the TLR9 signaling pathway, neuronal cells were exposed to melatonin, MDA (Figure 6) and LDH assay results (Figure 2) indicated that oxidative stress and cytotoxicity were significantly alleviated. Meanwhile, the expressions of γH2A.X protein and 8-OHdG were significantly decreased in these groups (Figure 6b–d). Further assessment of relevant TLR9 pathway proteins revealed significant reductions in the expression of TLR9 and its downstream effector c-Fos (Figure 6c–f). These results indicate that oxidative stress contributes to DNA damage, and inhibiting ROS production can mitigate PS-NPs-induced DNA damage. Notably, the levels of serotonin and NMDAR in neurons after melatonin supplementation did not significantly differ from those in the control group (Figure 6c,g,h).

## 4. Discussion

Previous studies confirmed the neurotoxicity of PS-NPs, but the underlying mechanisms remain to be clarified. In particular, understanding of the mechanisms contributing to anxiety-like behaviors is still in the exploratory stage. To address this knowledge gap, we utilized WBOs to investigate the neurotoxicity of PS-NPs. WBOs undergo four developmental stages: EB formation, differentiation, expansion, and maturation. We successfully generated WBOs containing neurons, astrocytes, microglia, and neural progenitor cells. Observations of the structure and developmental characteristics of WBOs revealed that PS-NPs affect their development and disrupt the dark–light layering of the cerebral cortex. This finding appears to align with previous observations by some researchers [27] that nanoplastics interfere with cell differentiation and migration.

Next, we evaluated inflammatory responses and oxidative stress in WBOs. By detecting the pro-inflammatory cytokines IL-1β and TNF-α, as well as the oxidative stress marker CAT, our results demonstrated that PS-NPs induce significant inflammatory responses and oxidative stress in WBOs. IL-1β and TNF-α promote the expression of inflammation-related genes, such as chemokines and cell adhesion molecules. Increased expression of these genes recruits immune cells to sites of inflammation and triggers further inflammatory cascades [28]. CAT is a key antioxidant enzyme that decomposes hydrogen peroxide and mitigates oxidative stress. Meanwhile, oxidative stress enhances the production of ROS and reactive nitrogen species, leading to pathological processes including lipid peroxidation of cell membranes, DNA strand breaks, and protein oxidation, which in turn induce neuronal damage and apoptosis. Consistent with relevant reports, PS-NPs exposure induces inflammatory responses [10] and oxidative stress in the nervous system of zebrafish, resulting in abnormalities in zebrafish neurotransmitter levels.

**Figure 7:**
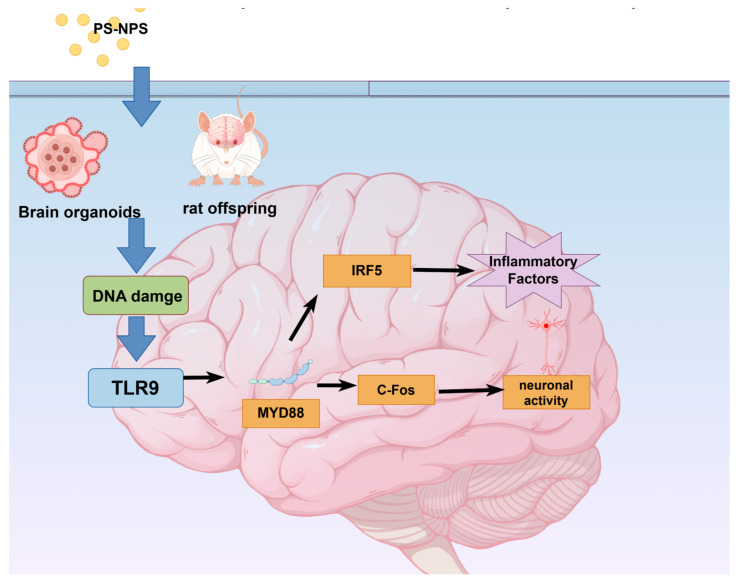
Mechanism of TLR9 activation Figure PS-NPs exposure can cause DNA damage, activate TLR9/MYD88 pathway and promote the release of inflammatory factors through IRF 5. Exposure of PS-NPs also triggers increased c-Fos expression, resulting in altered neural activity.

Literature reports have indicated that ROS generated by oxidative stress induce oxidative DNA damage [29,30]. Additionally, transcriptome-wide sequencing revealed significant enrichment of genes associated with DDR and the TLR9/MyD88 signaling pathway in WBOs. This finding provides a direction for further investigating the developmental neurotoxicity of PS-NPs. γH2A.X is a sensitive molecular marker for DNA damage and repair. Following DNA double-strand breaks, histone H2A.X is rapidly phosphorylated at Ser139 to form γH2A.X. The current results illustrated that PS-NPs induce oxidative stress in neurons, leading to intracellular DNA damage and a subsequent increase in γH2A.X levels [31], in line with reports by Ding [32] and Maity [33].The specific mechanistic changes are illustrated in Figure 7.

Consistent with previous findings, we observed that exposure of neonatal rats to PS-NPs resulted in epileptiform behavioral and brain structural changes, which might be related to alterations in DNA repair mechanisms. During embryonic development and neurogenesis, rapid cell proliferation and differentiation necessitate efficient DNA repair mechanisms to maintain genomic stability. The central nervous system is particularly sensitive to DNA repair defects, and unrepaired damage can lead to persistent DDR activation, which in turn triggers aberrant cell cycle reactivation, ultimately resulting in neuronal degeneration. Thus, DNA repair defects contribute to a wide range of neurodevelopmental and neurodegenerative diseases in humans [34]. In the nervous system, the role of TLR9 is closely related to DDR. TLR9 activates inflammatory signaling pathways in neurons by recognizing DNA double-stranded breaks and released histone fragments, thereby promoting the accumulation of DDR complexes. Additionally, we examined the expression of IRF5, which participates in neuroinflammation and enhances the pro-inflammatory response.

Following the recognition of double-strand breaks by TLR9, the MyD88-mediated signaling pathway is activated, which in turn upregulates c-Fos. c-Fos is an immediate early response gene that is closely associated with epileptic seizures [35]. Dragunow reported that the c-Fos gene is expressed in specific brain regions during epileptic seizures, possibly because of abnormal neuronal activity in these regions [36], as c-Fos serves as a marker of neuronal excitation [35]. Consistent with our results, Zhang et al. also confirmed that microplastic exposure induces epileptiform behaviors in neonatal rat [7] and zebrafish [37], and these behaviors were associated with c-Fos gene expression in the brain. Jin-Tao proposed that MyD88 could be a potential therapeutic target for epilepsy [38]. The elevation of MyD88 expression might be a key factor contributing to epileptiform behavior.

To verify excitotoxicity, we measured the levels of NMDAR, SYN1, and BDNF. PS-NPs impair synaptic plasticity and disrupt the balance between excitatory and inhibitory synapses, leading to abnormal neural activity. The ionotropic glutamate receptor NMDAR is a key excitatory neurotransmitter receptor. In the central nervous system, high levels of glutamate can induce neuronal death through NMDAR-mediated excitotoxicity [39]. Hyperactivity of NMDAR is associated with neuronal death in neurological disorders such as epilepsy, stroke, Alzheimer’s disease, and Parkinson’s disease. Blockade of NMDAR reduces neuronal death in the brain [39]. SYN1 regulates synaptic vesicle fusion and neurotransmitter release, processes that are critical for synapse formation and maintenance. Additionally, SYN1 is involved in the regulation of synaptic plasticity, which is essential for cognitive functions such as learning and memory [40]. BDNF plays a pivotal role in neuronal survival, synaptic plasticity, learning, and memory. Studies have revealed that reduced BDNF levels lead to decreased synaptic transmission efficiency and abnormal changes in synaptic architecture.

In previous studies, melatonin has been shown to reverse abnormally elevated oxidative stress and apoptosis [41,42]. After we intervened in PS-NPs-exposed neurons in vitro with melatonin, data demonstrated that melatonin treatment restored PS-NPs-induced DNA damage (Figure 6) and content of LDH (Figure 2) to near-normal levels. Notably, along with the alleviation of DNA damage, the levels of TLR9, c-Fos, and NMDAR also returned to levels close to those of the control group. This confirms that PS-NPs exposure induces neuronal DNA damage and activates the TLR9 signaling pathway, ultimately leading to neuronal excitotoxicity.

## 5. Conclusions

Our study suggests that exposure to PS-NPs can induce neurodevelopmental disorders. PS-NPs exposure activates the TLR9/MyD88 pathway, which triggers inflammation and the release of pro-inflammatory factors. Activation of the TLR9/MyD88 pathway also increases c-FOS levels, leading to altered neural activity. Furthermore, PS-NPs directly promote IRF5 expression via the TLR9 pathway, thereby inducing neuroinflammation. Our findings indicate that nanoplastics can be transferred across generations and affect the neurodevelopment of offspring. These results highlight the need to reconsider the environmental impact of plastic production and serve as a warning to environmental regulatory authorities to properly manage and handle plastic products. Collectively, our data demonstrate that PS-NPs may exert toxic effects on early human neurodevelopment. This study has certain limitations. Although we combined whole-transcriptome sequencing with WB validation, we did not verify the specific regulatory roles of microRNAs or RNA-binding proteins in mRNA-protein expression discrepancies. We will explore the underlying mechanisms in this regard in subsequent studies. We hope that our study will inform strategies for the prevention and control of microplastic pollution.

## Figures and Tables

**Table 1:** Antibody Usage Detail List.

Antibody	Dilution Ratio	Catalog Number	Supplier
SOX2	1:200	3579	CST
NeuN	1:200	Ab177487	Abcam
Olig2	1:200	Ab109186	Abcam
Tuj-1	1:200	4466S	CST
DAPI	1:200	CR2308091	Servicebio
Goat Anti-Mouse IgG (H+L) Alexa Fluor^®^ 555	1:500	4050-32	SBA
Goat Anti-Rabbit IgG (H+L) Alexa Fluor^®^ 488	1:500	1030-30	SBA
γH2A.X	1:400	AB303656	Abcam
TLR9	1:200	AF8193	CST
MyD88	1:200	23230-1-AP	Proteintech
C-FOS	1:50	YM3469	Immunoway
IRF5	1:200	10547-1-AP	Proteintech
FITC-labeled Goat Anti-Rabbit IgG (H+L)	1:200	A0562	Beyotime Biotechnology
TRITC-labeled Goat Anti-Rabbit IgG (H+L)	1:50	ZF-0316	ZsBio
Tubulin or β-tubulin	1:2000	10094-1-AP	Proteintech
NMDAR	1:500	AF6406	Affinity

## Data Availability

The original contributions presented in this study are included in the article. Further inquiries can be directed to the corresponding author.

## Abbreviations

The following abbreviations are used in this manuscript:

PS-NPs	Polystyrene nanoplastics
WBOs	whole-brain organoids
DDR	DNA damage response
TLR9	Toll-like receptor 9
8-OHdG	8-Hydroxy-2′-deoxyguanosine
CAT	Catalase
c-Fos	cellular oncogene Fos
IL-1β	Interleukin-1β
MLT	Melatonin
GSEA	Gene Set Enrichment Analysis
GO	Gene Ontology
KEGG	Kyoto Encyclopedia of Genes and Genomes
DSBs	Double-Strand Breaks
5-HT	Serotonin
